# Bayesian sample size determination in basket trials borrowing information between subsets

**DOI:** 10.1093/biostatistics/kxac033

**Published:** 2022-08-22

**Authors:** Haiyan Zheng, Michael J Grayling, Pavel Mozgunov, Thomas Jaki, James M S Wason

**Affiliations:** MRC Biostatistics Unit, University of Cambridge, Cambridge, CB2 0SR, UK and Population Health Sciences Institute, Newcastle University, Newcastle upon Tyne, NE2 4AX, UK; Population Health Sciences Institute, Newcastle University, Newcastle upon Tyne, NE2 4AX, UK; MRC Biostatistics Unit, University of Cambridge, Cambridge, CB2 0SR, UK; MRC Biostatistics Unit, University of Cambridge, Cambridge, CB2 0SR, UK and University of Regensburg, 93040 Regensburg, Germany; Population Health Sciences Institute, Newcastle University, Newcastle upon Tyne, NE2 4AX, UK

**Keywords:** Bayesian sample size determination, Borrowing strength, Master protocol, Mixture prior, Phase II

## Abstract

Basket trials are increasingly used for the simultaneous evaluation of a new treatment in various patient subgroups under one overarching protocol. We propose a Bayesian approach to sample size determination in basket trials that permit borrowing of information between commensurate subsets. Specifically, we consider a randomized basket trial design where patients are randomly assigned to the new treatment or control within each trial subset (“subtrial” for short). Closed-form sample size formulae are derived to ensure that each subtrial has a specified chance of correctly deciding whether the new treatment is superior to or not better than the control by some clinically relevant difference. Given prespecified levels of pairwise (in)commensurability, the subtrial sample sizes are solved simultaneously. The proposed Bayesian approach resembles the frequentist formulation of the problem in yielding comparable sample sizes for circumstances of no borrowing. When borrowing is enabled between commensurate subtrials, a considerably smaller trial sample size is required compared to the widely implemented approach of no borrowing. We illustrate the use of our sample size formulae with two examples based on real basket trials. A comprehensive simulation study further shows that the proposed methodology can maintain the true positive and false positive rates at desired levels.

## 1. Introduction

Clinical research in precision medicine (Mirnezami *and others*, 2012; Schork, 2015) continues to thrive as a consequence of the rapid technological advances for identifying possible prognostic and predictive disease factors at the genetic level (Aronson and Rehm, 2015; Morganti *and others*, 2019). Because of this, an increasing number of biomarker-driven therapies have been formulated. In oncology, for example, much attention has been paid to therapies targeting one or multiple genomic aberrations (Kim *and others*, 2011; Hyman *and others*, 2018; Redman *and others*, 2020). In contrast to conventional chemotherapy devised for treating histology-defined populations, such targeted therapies can potentially be beneficial to patients of various cancer (sub)types. Immune-mediated inflammatory diseases (IMIDs) (McInnes and Gravallese, 2021) are another area where targeted therapies can be useful (Grayling *and others*, 2021). IMIDs generally involve a clinically diverse group of conditions that share common underlying pathogenetic features, calling for the development of effective immune-targeted therapeutics (Pitzalis *and others*, 2020). This paradigm shift towards precision medicine has challenged the use of traditional one-size-fits-all approaches to trial design, which aim to estimate the population average treatment effect.

Master protocols (Woodcock and LaVange, 2017) comprise a class of innovative trial designs that address multiple investigational hypotheses. Newly emerging types include *basket trials* that can simultaneously evaluate a new treatment in stratified patient subgroups displaying a common disease trait (Renfro and Sargent, 2017; Tao *and others*, 2018). An implication of the stratification is that patients may respond very differently to the same treatment due to their distinct disease subtypes, stages, or status. Fully acknowledging the heterogeneity could lead to the use of stand-alone analyses that regard the stratified subgroups in isolation. Such an analysis strategy, though adopted in early basket trials, may not be ideal for realizing the promise of basket trials. This is mainly because it (i) fails to treat the combined (sub)trial components as a single study and (ii) often yields low-powered tests of the treatment effect, due to the small sample sizes.

Sophisticated analysis models, which feature *borrowing of information* between subgroups (ideally between those with commensurate treatment effects only), have been proposed in the statistical literature. One pivotal strategy is to fit a Bayesian hierarchical random-effects model (Thall *and others*, 2003; Berry *and others*, 2013), assuming that the subgroup-specific treatment effects are exchangeable, that is, as random samples drawn from a common normal distribution with unknown mean and variance. This methodology has been extended to involve (i) a finite mixture of exchangeability distributions (see, e.g., Liu *and others*, 2017; Chu and Yuan, 2018; Jin *and others*, 2020); as well as (ii) nonexchangeability distributions such that subgroups with an extreme treatment effect would not be over-represented (Neuenschwander *and others*, 2016). The former reflects the concern that some subsets of trial may be more commensurate between themselves than with others. A highly relevant proposal is to further cluster the subgroups so that the corresponding treatment effects are assumed to be exchangeable within the same cluster. Chen and Lee (2020) present a two-step procedure, by which subgroups are clustered using a Bayesian nonparametric model, before fitting an adjusted Bayesian hierarchical random-effects model.

A few authors have further recommended to discuss the exchangeability or commensurability of any two or more subgroups. These proposals are for better characterization of the complex trial data structure, which could involve mixtures of exchangeable or nonexchangeable patient subroups. Psioda *and others* (2021) apply a Bayesian model averaging technique (Hoeting *and others*, 1999) to accommodate the possibility that any configuration of subgroups may have the same or disparate response rate. Hobbs and Landin (2018) construct a matrix containing elements with a value of 0 or 1, indicating that any pair of subgroups can be exchangeable or nonexchangeable. Alternatively, Zheng and Wason (2022) propose measuring the pairwise (in)commensurability by distributional discrepancy to enable an appropriate degree of borrowing from each complementary subgroup, which yields a largest weight allocated to the most commensurate one(s).

Development of methods to choose an appropriate sample size for basket trials, however, appears to fall behind. A widely implemented approach is to sum up the sample sizes, calculated as if these trial subsets are to be carried out as separate studies. Whilst this could impair the efficiency of decision-making, alternative approaches to sample size determination that permits borrowing of information are lacking. In this article, we propose formal sample size planning for the design of basket trials. It strikes a balance between the sample size saving and the need of enrolling a sufficient number of patients to assure inferences about the subgroup-specific treatment effects. As the importance of randomized controlled trials has been increasingly emphasized in oncology (Ratain and Sargent, 2009; Grayling *and others*, 2019), IMIDs (Grayling *and others*, 2021) and rare-disease (Prasad and Oseran, 2015) research, this article will focus on randomized basket trial designs with a primary objective of simultaneously comparing the new treatment against control in various patient subgroups. We will thus develop our sample size formulae, presuming that the analysis is performed using a model adapted from Zheng and Wason (2022).

The remainder of this article is organized as follows. In Section 2, we introduce a Bayesian model that estimates the treatment effect specific to subgroups using the entire trial data, as well as the derivation of sample size formulae appropriate for basket trials. Two data examples are presented in Section 3 to illustrate the use of our formulae for the design of randomized basket trials. In Section 4, we describe a simulation study that evaluates the operating characteristics of randomized basket trials. Finally, we conclude with a brief discussion and highlight several areas that deserve future research in Section 5.

## 2. Methods

### 2.1. Leveraging complementary subtrial data into commensurate priors

Let us consider the design of a basket trial where patients can be classified into $K$ subgroups. These patients nonetheless share a common feature (e.g., a genetic aberration, clinical symptom, or mechanism of drug action), on which a new targeted therapy may potentially improve patient outcomes. Each component study in a distinct patient subgroup will hereafter be referred to as a trial subset (i.e., “subtrial” for short). Within each subtrial $k$, patients are randomized to receive either the experimental treatment (labeled $E$) with probability $R_k\in (0, 1)$, or a control (labeled $C$) with probability $(1 - R_k)$, for $k=1,\dots, K$. We further assume that the measured responses are normally distributed with their own subtrial-specific parameters: $X_{ijk} \sim N(\mu_{jk}, \sigma_{k}^2)$ with $i$ indexing patients, for $j = E, C; k = 1,\dots,K$. Letting $n_{k}$ denote the subtrial sample size, the difference in means is $\bar{X}_{Ek}-\bar{X}_{Ck}\sim N\left(\mu_{Ek}-\mu_{Ck}, \frac{\sigma_{k}^2}{n_kR_k(1-R_k)}\right)$. For the ease of notation, we let $\theta_k = \mu_{Ek} - \mu_{Ck}$ denote the treatment effect for subtrial $k$. It is important to clarify at the outset that this design aims to estimate the subtrial-specific treatment effects, that is, $\theta_1, \dots, \theta_K$, instead of an overall treatment effect averaged over all subtrials. If permitting borrowing of information across subtrials, these treatment effects are to be estimated using the entire trial data (with $\sum_{k=1}^K n_k$ patients) rather than in isolation (with $n_1, \dots, n_K$ patients, respectively).

We follow Zheng and Wason (2022) in specifying commensurate priors for each $\theta_k$, using information from the $(K-1)$ complementary subtrials indexed by $q\neq k, \, \forall k = 1, \dots, K$. This methodology regards any $\theta_q$ as a biased representation of $\theta_k$, yet the direction and the size of such bias are unknown (Hobbs *and others*, 2011). More specifically, these commensurate priors are formulated as conditional normal distributions that are centered at $\theta_q$s, respectively; whilst the precisions (i.e., reciprocal of variances), denoted by $\nu_{qk}$, accommodate the heterogeneity between two subtrials $k$ and $q$. Our commensurate prior models for the continuous location parameter $\theta_k$ can thus be given by


(2.1) \begin{equation*}
\begin{split}
\theta_{k} \mid \theta_q, \nu_{qk} & \sim N(\theta_q, \nu_{qk}^{-1}), \qquad \forall k = 1, \dots, K, \\
\nu_{qk} & \sim w_{qk} \text{Gamma}(a_{1}, b_{1}) + (1-w_{qk})\text{Gamma}(a_{2}, b_{2}),
\end{split}
\label{eq:commenprio}
\end{equation*}

where a two-component Gamma mixture prior (with $a_1/b_1$ and $a_2/b_2$ being the respective means of the component distributions), instead of a spike-and-slab prior in the original proposal, is placed on each $\nu_{qk}$ for the convenience of analytic tractability (Zheng *and others*, 2022). In particular, these two Gamma mixture components correspond to extreme cases of substantial or limited discounting of information from a complementary subtrial $q$. For illustration, we suppose that the first Gamma mixture component has its density massively on small values, and the second component on large values. The prior mixture weight $w_{qk}\in [0, 1]$, which plays a role of balancing between the extreme cases, can thus reflect one’s preliminary skepticism about the degree of commensurability between $\theta_k$ and $\theta_q$. That is, when subtrials $k$ and $q$ are thought of as incommensurate (commensurate), $w_{qk}$ can be set close to 1 (0), thus forcing the conditional prior variance $\nu_{qk}^{-1}$ towards large (small) values for substantial (limited) discounting.

By integrating out $\nu_{qk}$, the conditional prior for $\theta_{k}$ given $\theta_q$ only follows a shifted and scaled $t$ mixture distribution, with its two components both centered at $\theta_q$. This unimodal $t$ mixture distribution can further be approximated by matching the first two moments to give


(2.2) \begin{equation*}
\theta_k \mid \theta_q \, \dot\sim \, N\left(\theta_q, \frac{w_{qk}b_{1}}{a_{1}-1} +\frac{(1-w_{qk})b_{2}}{a_{2}-1} \right), \quad \text{ with } a_{1}, a_{2} > 1,
\label{eq:margmui}
\end{equation*}

which incorporates the respective variances of the $t$ component distributions. As has been shown by Zheng *and others* (2022), this normal approximation provides good properties for the coverage of credible intervals of interest. Note that the location of each commensurate prior, $\theta_q$, is an unknown parameter. It captures the information from a complementary subtrial $q$, of which the required sample size $n_q$ as well as the allocation proportion $R_q$ is yet to be determined.

Let $\boldsymbol{x}_q = \{x_{1Eq}, \dots, x_{n_qEq}; x_{1Cq}, \dots, x_{n_qCq} \}$ denote the data of a complementary subtrial $q$. We consider the difference of sample means, $\bar{X}_{qE}-\bar{X}_{qC}$, as the random variable to draw the Bayesian inference. With an “uninformative” operational prior $\theta_q\sim N(m_{0q}, s_{0q}^2)$, we derive the posterior as


(2.3) \begin{equation*}
\begin{split}
\theta_q \mid \boldsymbol{x}_q &\sim N\left(\lambda_q, \left(\frac{1}{s_{0q}^2} + \frac{n_qR_q(1-R_q)}{\sigma_{q}^2}\right)^{-1} \right), \\
\text{with } \qquad \lambda_q &= \frac{m_{0q}}{1+ \frac{s_{0q}^2}{\sigma_{q}^2}\cdot n_qR_q(1-R_q)}+\frac{\bar{x}_{Eq} - \bar{x}_{Cq}}{1+\frac{\sigma_{q}^2}{s_{0q}^2}\cdot\left[n_qR_q(1-R_q)\right]^{-1}}{,}
\end{split}
\label{eq:oppost}
\end{equation*}

where $\bar{x}_{jq}$ denotes the average response of samples by treatment group $j = E, C,$ within subtrial $q$. Combining (2.2) and (2.3), we obtain a commensurate prior for $\theta_k$ that leverages data of a complementary subtrial $q\neq k$:


(2.4) \begin{equation*}
\begin{split}
\theta_k \mid \boldsymbol{x}_q &\sim N(\lambda_q, \xi_{qk}^2), \qquad \forall k = 1, \dots, K, \\
\text{with } \qquad \xi_{qk}^2 &= \left(\frac{1}{s_{0q}^2} + \frac{n_qR_q(1-R_q)}{\sigma_{q}^2} \right)^{-1} + \frac{w_{qk}b_{1}}{a_{1}-1} +\frac{(1-w_{qk})b_{2}}{a_{2}-1}.
\end{split}
\label{eq:borrowprior}
\end{equation*}

Consider now borrowing information from all complementary subtrials, with $K \geq 3$. Let $\boldsymbol{x}_{(-k)}$ denote the data from all subtrials excluding $k$, that is, all the $(K-1)$ sets of complementary data for subtrial $k$. By the convolution operator (Grinstead and Snell, 1997), we stipulate a collective, commensurate prior for leveraging all complementary subtrial data:


(2.5) \begin{equation*}
\theta_k \mid \boldsymbol{x}_{(-k)} \sim N\left(\sum_{q\neq k} p_{qk} \lambda_q, \sum_{q\neq k} p_{qk}^2 \xi_{qk}^2 \right), \qquad \forall k = 1, \dots, K,
\label{eq:collprior}
\end{equation*}

where $p_{qk}$ are the synthesis weights, with $\sum_q p_{qk} = 1$, assigned to the respective commensurate priors specified using $\boldsymbol{x}_q$. These synthesis weights can be transformed from the chosen values for $w_{qk}$ in the commensurate prior models, following an objective-directed approach (Zheng and Wason, 2022). More specifically, we expect the largest synthesis weight, $p_{qk}$, is assigned to the most commensurate prior $N(\lambda_q, \xi_{qk}^2)$, specified based on a subtrial $q\neq k$ that manifests the smallest discrepancy with subtrial $k$ out of all the $(K-1)$ complementary subtrials. Recall each $w_{qk}$, as one key parameter to determine $N(\lambda_q, \xi_{qk}^2)$, would have been chosen to appropriately reflect the pairwise discrepancy (i.e., incommensurability). One may apply a decreasing function of $w_{qk}$ to compute $p_{qk}$. A $K\times K$ matrix can be constructed to contain all $w_{qk}$ in column $k$ and row $q$ as:


$$
\begin{pmatrix}
0 & w_{12} & \cdots & w_{1K} \\
w_{21} & 0 & \cdots & w_{2K} \\
\vdots & \vdots & \ddots & \vdots \\
w_{K1} & w_{K2} & \cdots & 0
\end{pmatrix}.
$$

We note that this matrix should be symmetric with $w_{qk} = w_{kq}$, since each is intended to reflect the level of pairwise incommensurability. That is, the magnitude of incommensurability between subtrials $k$ and $q$ is the same as that between $q$ and $k$. If stratifying the matrix by column, the off-diagonal elements in column $k$ represent the postulated levels of discounting with respect to the complementary subtrial data. Recall that the latter has been used to specify the respective commensurate priors in the form of (2.4), with $q\neq k$. The decreasing function given by


$$
p_{qk} = \frac{\exp(-w_{qk}^2/c_0)}{\sum_q \exp(-w_{qk}^2/c_0)}, \qquad \forall k = 1, \dots, K,
$$

has been illustrated to have satisfactory properties (Zheng and Wason, 2022; Zheng *and others*, 2022). The concentration parameter $c_0$, if set equal to a value close to $0^+$, appropriately discerns the $(K-1)$ values of $w_{qk}$; thus, a $p_{qk}\rightarrow 1$ would be assigned to the corresponding commensurate prior for $\theta_k$ based on $\boldsymbol{x}_q$, in which the smallest $w_{qk}$ has been used. Otherwise, a value of $c_0\gg w_{qk}$ yields nearly all $p_{qk}$ to equal $1/(K-1)$ irrespective of the values of $w_{qk}$. Moreover, this transformation yields equal $p_{qk}$ when all $w_{qk}$ are equal. We generally recommend setting $c_0$ to a value that is substantially smaller than the magnitude of $w_{qk}$; for a thorough evaluation of performance by varying $c_0$, we refer the reader to Zheng and Wason (2022).

By using Bayes’ Theorem, the collective commensurate prior in the form of (2.5) will be updated by the contemporary subtrial data $\boldsymbol{x}_k$ to give the posterior distribution as


(2.6) \begin{equation*}
\theta_k \mid \boldsymbol{x}_k, \boldsymbol{x}_{(-k)} \sim N \left(d_{\theta_k},
\left(\frac{1}{\sum_q p_{qk}^2 \xi_{qk}^2} + \frac{n_kR_k(1-R_k)}{\sigma_{k}^2} \right)^{-1}
\right).
\label{eq:collpost}
\end{equation*}

The posterior mean is a convex combination of the prior mean $\sum p_{qk}\lambda_q\, (q \neq k)$ and the data likelihood. We will give the exact expression of $d_{\theta_k}$ in Section 4 to carry out the decision-making for simulated trials.

### 2.2. Sample size formulae for basket trials comparing two normal means

The frequentist approach to sample size determination makes use of hypothesis testing, with $H_{0k}: \theta_k \leq 0$ against $H_{1k}: \theta_k > 0$, if assuming that greater values of $X_{ijk}$ indicate better effect. In this traditional framework, a study sample size is computed such that the treatment effect, $\theta_k$, will be found significant at a level $\alpha$ with probability $1-\beta$, given a certain magnitude of the treatment effect considered clinically meaningful.

We follow the Bayesian decision-making framework, presented by Whitehead *and others* (2008), to compute two interval probabilities from our posterior distribution as derived in (2.6), so that the subtrial sample sizes, $n_1, \dots, n_K$, are sought for providing compelling evidence of $E$ being *either* superior to *or* not better than $C$ by some magnitude $\delta$ in each subtrial $k = 1, \dots, K$. The posterior distribution of $\theta_k$ specific to each subtrial $k=1, \dots,K$ will thus be evaluated to declare that $E$ is


(2.7) \begin{align*}
\text{(i) efficacious, if} \quad & \mathbb{P}(\theta_k > 0 \mid \boldsymbol{x}_k, \boldsymbol{x}_{(-k)}) \geq \eta{,} \label{eq:criterion1} \\
\end{align*}

(2.8) \begin{align*}
\text{(ii) futile, if} \quad & \mathbb{P}(\theta_k \leq \delta \mid \boldsymbol{x}_k, \boldsymbol{x}_{(-k)}) \geq \zeta{,} \label{eq:criterion2}
\end{align*}

where $\eta$ and $\zeta$ are probability thresholds for the success and futility criteria, respectively. By using this decision rule, the two posterior tail probabilities of $\theta_k$ are controlled. Specifically, we desire the area under the density from $-\infty$ to the left of 0 to be limited below $1-\eta$, with the area under the density from $\infty$ to the right of $\delta$ to be below $1-\zeta$. The sample size therefore needs to be sufficiently large for a decisive declaration of the treatment effectiveness or futility per subtrial $k$. That is, $d_{\theta_k}/\sigma_{\theta_k} \geq z_\eta$ or $(\delta - d_{\theta_k})/\sigma_{\theta_k} \geq z_\zeta$ should be guaranteed. Here, $z_\eta$ satisfies $\Phi(z_\eta) = \eta,$ where $\Phi(\cdot)$ denotes the standard normal distribution function, with $z_{\zeta}$ defined similarly. We thus require $\delta/\sigma_{\theta_k} \geq z_\zeta + z_\eta$, which leads to


(2.9) \begin{equation*}
\frac{1}{\sigma_{\theta_k}^2} \geq \left( \frac{z_\eta + z_\zeta}{\delta}\right)^2.
\label{eq:SSbase}
\end{equation*}

The left-hand side of (2.9) is precisely the posterior precision for $\theta_k$.

When borrowing of information is not permitted, $\mathbb{P}(\theta_k >0 \mid \boldsymbol{x}_k)$ and $\mathbb{P}(\theta_k \leq \delta \mid \boldsymbol{x}_k)$ are computed instead. Thus, $\frac{1}{\sigma_{\theta_k}^2} = \frac{1}{s_{0k}^2} + \frac{n_kR_k(1-R_k)}{\sigma_{k}^2}$, which can be rearranged to give


(2.10) \begin{equation*}
n_k^{0} \geq \frac{\sigma_{k}^2}{R_k(1-R_k)}\left[\left(\frac{z_\eta + z_\zeta}{\delta}\right)^2 - \frac{1}{s_{0k}^2} \right], \quad \forall k = 1, \dots, K.
\label{NoBrwSSD}
\end{equation*}

By contrast, based on the proposed Bayesian model for borrowing of information, $\sigma_{\theta_k}^2$ comes from the closed-form expression in (2.6). This leads to


(2.11) \begin{equation*}
n_k \geq \frac{\sigma_{k}^2}{R_k(1-R_k)}\left[\left(\frac{z_\eta + z_\zeta}{\delta}\right)^2 - \frac{1}{\sum_{q} p_{qk}^2 \xi_{qk}^2} \right], \quad \forall k = 1, \dots, K, \; q\neq k,
\label{eq:BayesSSD}
\end{equation*}

which looks similar to (2.10), but involves the commensurate prior variances $\xi_{qk}^2$ in the form of (2.4). The latter leverages the complementary subtrial information. Thus, a smaller integer for $n_k$ could be expected if the complementary subtrials, labeled $q\neq k$, are to collect rich information and, further, considerable borrowing of information happens. To ensure the inference in all $K$ subtrials, we require that $\forall k = 1, \dots, K$,


\begin{equation*}
\frac{n_kR_k(1-R_k)}{\sigma_{k}^2} + \left[\sum_q p_{qk}^2 \left(\left(\frac{1}{s_{0q}^2} + \frac{n_qR_q(1-R_q)}{\sigma_{q}^2} \right)^{-1} + \frac{w_{qk}b_{1}}{a_{1}-1} +\frac{(1-w_{qk})b_{2}}{a_{2}-1} \right)\right]^{-1} \geq \left(\frac{z_\eta + z_\zeta}{\delta}\right)^2,
\end{equation*}

with $p_{qk}$ transformed from $w_{qk}$ following the stipulation in Section 2.1. The $K$ nonlinear equations of $n_k$ and its $n_q$s are continuously differentiable. We apply Newton’s method for systems of nonlinear equations (Dennis and Schnabel, 1983) to find $n_1, \dots, n_K$ that satisfy the $K$ constraints simultaneously, given known $w_{qk}$ and $\sigma_k^2$.

The importance of $w_{qk}$, in computing subtrial sample sizes is of particular interest; see Figure S1 of the Supplementary material available at *Biostatistics* online for the illustration for a special case. Whilst the definition of data (in)commensurability can vary on a case-by-case basis, these values may better be specified in collaboration with a subject-matter expert. Those conversations may also help quantify the magnitude of incommensurability, particularly in the absence of pilot data or relevant investigation. We caution that the choice of $w_{qk}$ should reflect the level of pairwise (in)commensurability *a priori* in the practical implementation, rather than the desire to obtain a minimal sample size.

## 3. Application to the design of randomized basket trials

### 3.1. A UK-based basket trial for treating patients with chronic diseases

The randomized, placebo-controlled Obeticholic acid for the Amelioration of Cognitive Symptoms trial (known as the “OACS trial”, ISRCTN15223158) aims to assess the efficacy of Obeticholic acid ($E$), as compared to a placebo ($C$), for treating cognitive deficits. The OACS trial is split into three subtrials, each focusing on a distinct patient subpopulation defined by the disease stage and clinical area. Namely, OACS-1 for patients with stabilized primary biliary cholangitis (PBC) $>2$ years since diagnosis; OACS-2 for patients with new-onset PBC $\leq 2$ years; and OACS-3 for patients with Parkinson’s disease. The primary outcome is a composite cognitive test score obtained from the CANTAB platform (Goldberg, 2013), which is an extensively used tool in clinical practice. The reduction in the composite CANTAB score from the baseline, after 26 weeks of treatment, will be analyzed as a normally distributed primary endpoint. We assume the magnitude of such reduction can be adequately depicted by values ranging from $-$5 to 5, where a high value suggests the improvement of cognitive symptoms in a patient.

The sample sizes have been determined as 40 (20 on $E$ and $C$ each) for OACS-1, 25 (15 on $E$ and 10 on $C$) for OACS-2, 25 (15 on $E$ and 10 on $C$) for OACS-3, assuming that these subtrials are to be analyzed on their own. As specified in the trial protocol, these were not computed based on hypothesis testing considerations originally. However, the resulting sample sizes are consistent with $\sigma_1^2 = 6.177,\, \sigma_2^2 = 5.134,\, \sigma_3^2 = 5.134$ to ensure 90$\%$ statistical power for OACS-1 and 80$\%$ for the other subtrials to detect a difference of $\delta = 2.3$, whilst controlling the type I error rate below 0.05 at the subtrial level. In the following, we use these quantities to illustrate the application of the proposed methodology, if the OACS basket trial would have been designed using the Bayesian decision-making framework outlined above.

We set $s_{0k}^2 = 100$ and $\nu_{qk} \sim w_{qk} \text{Gamma}(1.1, 1.1) + (1-w_{qk})\text{Gamma}(54, 3)$ for all $k = 1, \dots, 3$. Specifically, the two Gamma mixture components correspond to the mean and a 95$\%$ credible interval for each $\nu_{qk}$ as 1 [0.041, 4.286] and 18 [13.522, 23.108], respectively. This accommodates two extreme cases for limited or substantial borrowing when $w_{qk} = 1$ or 0.

If no borrowing is permitted, $n_k^0 = 39.8,\, 24.8,\, 24.8$ according to (2.10). This is about the same as the actual sample sizes of 40, 25, 25, respectively. For illustration purposes only, we use the following matrix of $w_{qk}$


$$
\begin{pmatrix}
w_{11} & w_{12} & w_{13} \\
w_{21} & w_{22} & w_{23} \\
w_{31} & w_{32} & w_{33} \\
\end{pmatrix} =
\begin{pmatrix}
0 & 0.239 & 0.417 \\
0.239 & 0 & 0.145 \\
0.417 & 0.145 & 0
\end{pmatrix},
$$

and $c_0 = 0.05$ to compute the synthesis weights $p_{qk}$, following the objective-directed approach outlined in Section 2.1. The subtrial sample sizes are found to be $n_k = 33.3, \, 11.8,\, 18.2$, fixing $\eta = 0.95$ and $\zeta = 0.90$ for subtrial 1 or 0.80 for subtrial 2 maintaining the same treatment allocation ratios $R_k = 0.5, \, 0.6, \, 0.6$. These are considerably smaller than the subtrial sample sizes assuming no borrowing or the frequentist counterparts.

### 3.2. Simultaneous evaluation of a new inhibitor in seven cancer subtypes

The ongoing SUMMIT basket trial (NCT01953926) adopted a single-arm design to evaluate a new pan-HER kinase inhibitor neratinib in 141 patients with HER2-mutant or HER3-mutant tumors (Hyman *and others*, 2018). A binary outcome (i.e., responder or no responder, corresponding to a tumor shrinkage $\geq$ 30$\%$ or below) was used in line with the RECIST criteria (Eisenhauer *and others*, 2009). The SUMMIT trial additionally reported the analysis on secondary outcomes, which include the change in tumor volume on a continuous scale of $-$100$\%$ to 100$\%$. We assume a new randomized basket trial would follow, wherein the change in tumor volume from $-$100$\%$ to 100$\%$ is the primary outcome. A negative sign indicates clinical benefit since it is hoped that the tumor shrinks from the baseline measurement due to the treatment. With $\delta < 0$, the trial decision criterion expressed by (2.7) and (2.8) should be altered as (i) efficacious, if $\mathbb{P}(\theta_k < 0 \mid \boldsymbol{x}_k, \boldsymbol{x}_{(-k)}) \geq \eta$ and (ii) futile, if $\mathbb{P}(\theta_k \geq \delta \mid \boldsymbol{x}_k, \boldsymbol{x}_{(-k)}) \geq \zeta$.

We narrow the focus on seven of the originally investigated 21 cancer subtypes only, of which the mean responses for patients receiving neratinib ($E$) were approximately $\mu_{Ek} = -0.489, 0.226$, $-0.181, 0.293$, $0.329, -0.275, -0.136$. We further assume that the mean responses on a control treatment embedded in the newly planned basket trial are $\mu_{Ck} = 0$, and that patients within each subtrial have equal probability to receive $E$ or $C$; that is, $R_k = 0.5$ for $k = 1, \dots, 7$.

Based on the published SUMMIT trial results (Hyman *and others*, 2018), we assume that the subtrial-specific variances are $\sigma_{k}^2 = 0.587^2$, $0.345^2,\, 0.380^2,\, 0.347^2,\, 0.344^2,\, 0.392^2,\, 0.392^2$. The pairwise discrepancy between the assumed outcome distributions, $N(\mu_{Ek}, \sigma_{k}^2)$, can be quantified using, for example, the Hellinger distance (Dey and Birmiwal, 1994),


$$
w_{qk} = \left[1 - \sqrt{\frac{2\sigma_q \sigma_k}{\sigma_q^2 + \sigma_k^2}}\exp \left( -\frac{(\mu_{Eq} - \mu_{Ek})^2}{4(\sigma_q^2 + \sigma_k^2)} \right) \right]^{1/2}.
$$

By targeting $\delta = -0.40$ and retaining the specification of other parameters from Section 3.1, $n_k = 52.0, \, 17.3, \, 20.5, \, 17.0, \, 17.1, \, 22.5, \, 22.0$. If no borrowing is permitted, $n_k^0 = 53.3, \, 18.4, \, 22.3, \, 18.6$, $18.3, \, 23.8, \, 23.8$. Only a small reduction in the sample sizes has been observed, largely because most of the values of $w_{qk}$ are above 0.30.

## 4. Simulation study

### 4.1. Basic setting

**Fig. 1.**
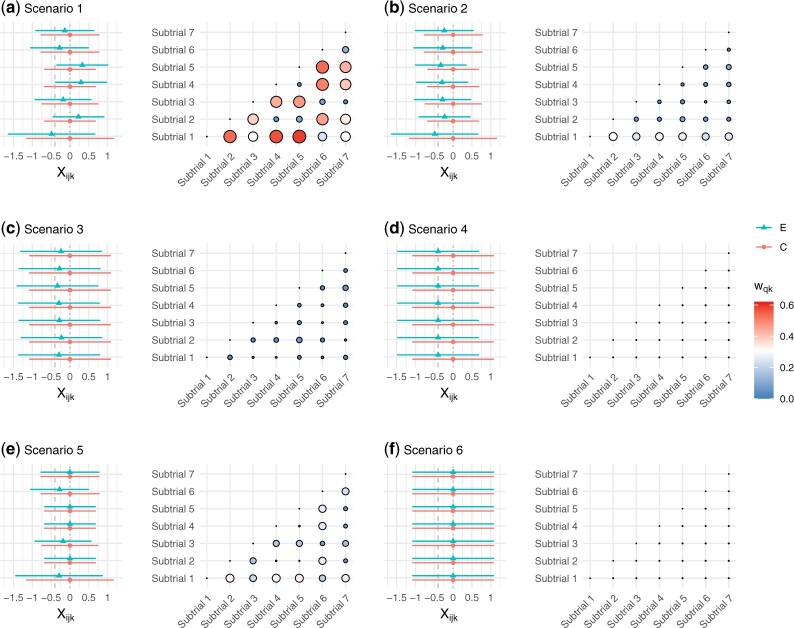
Simulation scenarios depicted by the distributions of $X_{ijk}, j = E, C, \, k = 1, \dots, 7$, along with the visualization of $w_{qk}$ for pairwise discounting of incommensurate complementary subtrial data. The green triangle and red circle mark the normal means $\mu_{Ek}$ and $\mu_{Ck}$, respectively; the endpoints of each horizontal bar are computed by $\mu_{jk}\pm 1.96\sigma_k^2$. The dashed and dotted vertical lines indicate the effect size of $\delta$ and 0, respectively. This figure appears in colour in the electronic version of this article, and any mention of colour refers to that version.

Motivated by the SUMMIT trial, we consider the sample size planning of basket trials following the same data structure. That is, the basket trial would enroll $n_1, \dots, n_7$ patients to the respective subtrials, with $R_k = 0.5$ in all $K = 7$ subgroups, under six possible scenarios. Figure 1 visualizes the six simulation scenarios, where the location and length of lines suggest the distributions of $X_{ijk}, \, j = E, C$, while a larger bubble corresponds to a larger value of $w_{qk}$. Here, we have followed the specification of $w_{qk}$ given in Section 3.2 to compute the pairwise Hellinger distance to characterize (in)commensurability and obtain $w_{qk}$ for the levels of borrowing/discounting strength. Scenarios 4 and 6 correspond to two special cases of the treatment being consistently effective (alternative hypotheses) and consistently futile (null hypotheses), respectively. Both scenarios feature perfect commensurability; that is, the outcomes $X_{iEk}$ and $X_{iCk}$ have their respective, same distribution across subtrials, so all $w_{qk} = 0$. Scenario 5 represents a mixed null situation, where $\theta_k = 0$ holds for four of the subtrials only. The other scenarios are constructed to reflect various levels of data incommensurability. Exact configurations of these simulation scenarios, that is, values of $\mu_{Ek}$ along with all $\mu_{Ck} = 0$ as well as the subtrial-specific variances $\sigma_k^2$, are listed in Table S1 of the Supplementary material available at *Biostatistics* online.

We retain the prior specification and the probability thresholds unchanged from Section 3. Table 1 thus gives the subtrial sample sizes required to reach a decisive conclusion of either $E$ is superior to or not better than $C$ by $\delta = -0.4$ using the respective sample size formulae. Because no $w_{qk}$ has been set to 1 in any scenario, the sample sizes $n_k$ computed from the approach of borrowing are generally smaller than $n_k^0$ from the approach of no borrowing. Scenario 1 was constructed from the illustrative data example in Section 3.2. Since relatively large values have been chosen for $w_{qk}$, only a slight decrease in sample sizes is observed. Unlike Scenario 1 that displays divergent effects, Scenario 2 is featured with a higher degree of commensurability that $E$ has an enhanced benefit over $C$ in all subtrials. A smaller trial sample size is then sufficient. Scenario 3 has all the variances $\sigma_k^2 = 0.3$. Consequently, $n_k^0$, based on the approach of no borrowing, are solved to be equal to 46.4 for all subtrials. Whereas, using the proposed methodology, the sample sizes for subtrials 1, 3, 4, and 6 are smaller, as these are recognized to be more commensurate between themselves than with the other three. A similar explanation can be given to Scenario 5: subtrials 2, 4, and 5 have greater sample size savings because the corresponding $w_{qk}$ takes smaller values. Scenarios 4 and 6 represent the situations of perfect commensurability. With all $w_{qk} = 0$, a substantial reduction in the subtrial sample sizes is observed.

**Table 1.** The required subtrial sample sizes, computed using the proposed methodology or the approach of no borrowing, to detect a difference of $\delta = -0.4$, when setting $\eta = 0.95, \zeta = 0.80$

		Required subtrial sample size							
		$k = 1$	$k = 2$	$k = 3$	$k = 4$	$k = 5$	$k = 6$	$k = 7$	Total
Scenario 1	$n_k^0$ (No borrowing)	53.2	18.4	22.3	18.6	18.3	23.7	23.7	178.2
	$n_k$ (Proposed)	52.0	17.3	20.5	17.0	17.1	22.5	22.0	168.4
Scenario 2	$n_k^0$ (No borrowing)	53.2	18.4	22.3	18.6	18.3	23.7	23.7	178.2
	$n_k$ (Proposed)	50.6	15.7	17.4	15.1	15.6	18.9	19.6	152.9
Scenario 3	$n_k^0$ (No borrowing)	46.4	46.4	46.4	46.4	46.4	46.4	46.4	324.8
	$n_k$ (Proposed)	23.3	32.0	22.6	24.4	32.9	23.3	30.2	188.7
Scenario 4	$n_k^0$ (No borrowing)	46.4	46.4	46.4	46.4	46.4	46.4	46.4	324.8
	$n_k$ (Proposed)	8.9	8.9	8.9	8.9	8.9	8.9	8.9	62.3
Scenario 5	$n_k^0$ (No borrowing)	53.2	18.4	22.3	18.6	18.3	23.7	23.7	178.2
	$n_k$ (Proposed)	50.8	14.3	20.4	14.5	14.2	22.1	20.7	157.0
Scenario 6	$n_k^0$ (No borrowing)	46.4	46.4	46.4	46.4	46.4	46.4	46.4	324.8
	$n_k$ (Proposed)	8.9	8.9	8.9	8.9	8.9	8.9	8.9	62.3

In the numerical evaluation below, we simulate the outcomes $X_{ijk}, j = E, C,$ from $N(\mu_{Ek}, \sigma_k^2)$ and $N(\mu_{Ck}, \sigma_k^2)$ for patient $i = 1, \dots, n_k,$ within subtrial $k = 1, \dots, 7$. For each scenario, 100 000 replicates of the basket trials are simulated to fit:

- the proposed Bayesian model, which yields the posterior distributions for $\theta_k$ in the form of (2.6), with
  $$
d_{\theta_k} = \frac{\sigma_k^2/(n_kR_k(1-R_k))\cdot \sum_q p_{qk}\lambda_q + (\bar{x}_{Ek} - \bar{x}_{Ck}) \cdot \sum_q p_{qk}^2 \xi_{qk}^2}{\sum_q p_{qk}^2 \xi_{qk}^2 + \sigma_k^2/(n_kR_k(1-R_k))},
$$
- a Bayesian stand-alone analysis model for no borrowing of information. Operational priors, that is, $N(m_{0k}, s_{0k}^2)$, are placed on each $\theta_k$. This leads to the posterior distributions for $\theta_k$ based on $\boldsymbol{x}_k$ alone, which has the same form as (2.3), with the subscript $q$ replaced by $k$. We set all $m_{0k} = 0$ in the simulations.

### 4.2. Results

**Fig. 2.**
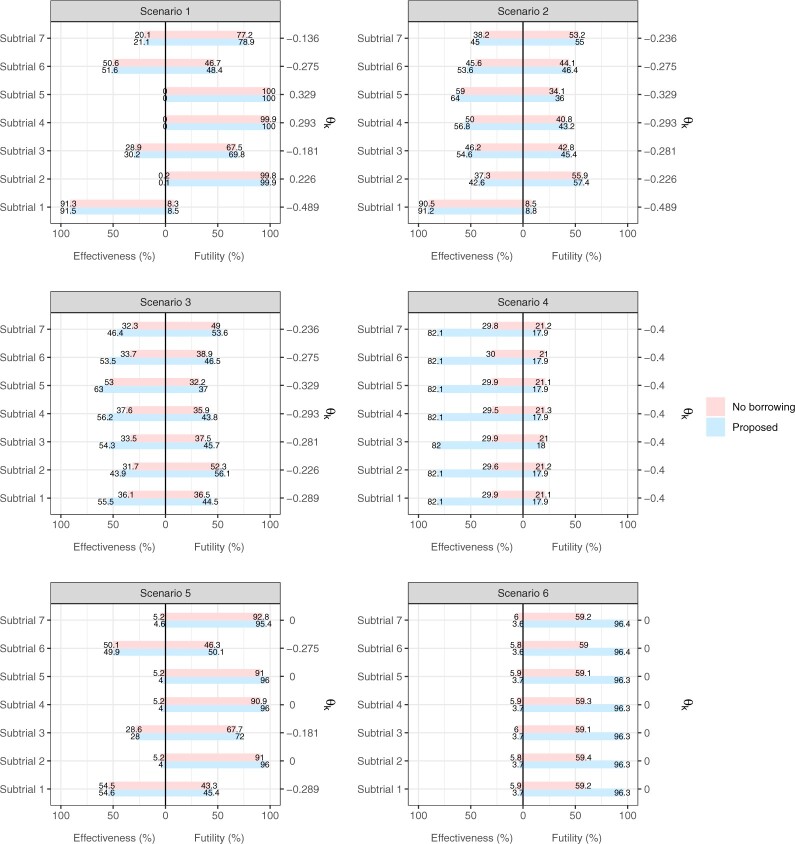
Percentage of (sub)trials that conclude $E$ is efficacious (the left half of each plot) or not better than $C$ by $\delta = -0.4$, that is, observing a shrinkage of 40$\%$ in the tumor volume (the right half of each plot). True subtrial-specific treatment effects, $\theta_k = \mu_{Ek} - \mu_{Ck}$, have been indicated in a second $y$-axis.

We summarize the frequency of simulated trials concluding that $E$ is either efficacious or futile, based on the 100 000 replicates per scenario and model. Figure 2 depicts the percentages of (sub)trials declaring effectiveness of $E$ and those declaring futility. Wherever the lengths of bars sum up to 100$\%$, this means the study is planned with an adequate sample size for decisive decision-making. As we can observe, collecting data from $n_1, \dots, n_7$ patients to fit the proposed analysis model ensures 100$\%$ of the (sub)trials to conclude that $E$ is either superior to or not better than $C$ by $\delta$. Whereas, it is not the case (i.e., all below 100$\%$) if implementing the Bayesian model for no borrowing, since larger sample sizes (i.e., $n_k^0$ in Table 1) would be required to ensure the same level of posterior distribution informativeness for the trial decision. In Scenarios 1 and 2 where $n_k$ and $n_k^0$ are comparable, these two Bayesian models yield comparable proportions of (sub)trials with a decisive trial decision. Yet in Scenarios 4 and 6, where substantial sample size savings are made, a disparity is observed, because the posterior distributions for $\theta_k$ based on $\boldsymbol{x}_k$ alone are far less informative than those based on $\boldsymbol{x}_1, \dots, \boldsymbol{x}_K$.

In Scenarios 2 and 3, $E$ is potentially superior to $C$ yet the magnitude tends to be smaller than desired on average. Only subtrial 1 has a mean treatment effect greater than $\delta$, so about 91.2$\%$ of the simulated (sub)trials have declared $E$ being effective. By contrast, subtrials 2 and 5 have mean treatment effects closest to 0 and $\delta$, respectively. Therefore, subtrial 2 has higher chance to declare futility than the effectiveness of $E$, but subtrial 5 is on the contrary. Scenario 4 mimics the borderline case where the mean treatment effect just has the size of $\delta$. Using the proposed methodology, about 82.1$\%$ of the simulated (sub)trials have favored $E$ for effectiveness in all seven subtrials. These subtrialwise true positive rates are about equal to our chosen threshold $\zeta = 0.80$. Scenario 5 assumes a mixture of subtrial-specific treatment effects with $\theta_k = 0$ or $\geq \delta$. Referring to subtrials 2, 4, 5, and 7, less than 5$\%$ of the simulated trials conclude effectiveness erroneously. The two Bayesian models yield similar operating characteristics in this scenario, as the computed $n_k$ and $n_k^0$ were close. In Scenario 6, the proportion of incorrect decision of effectiveness is maintained below 5$\%$ for all subtrials using the proposed methodology. Unsurprisingly, using the approach of no borrowing to analyze the basket trial from only 62.3 patients has a much lower chance of obtaining a definitive conclusion. The overall false positive rate (i.e., probability of incorrectly rejecting at least one subtrial that has true $\theta_k = 0$), based on the proposed methodology, is 0.150 for Scenario 5 and 0.054 for Scenario 6. These increase to 0.192 and 0.346, respectively, if the approach of no borrowing is implemented instead. This is unsurprising because the sample sizes have been computed to control the error rate at the subtrial level. For strong control of the overall error rate, multiplicity adjustment such as the Bonferroni procedure is required. After the correction, one can still expect a benefit from borrowing information.

**Fig. 3.**
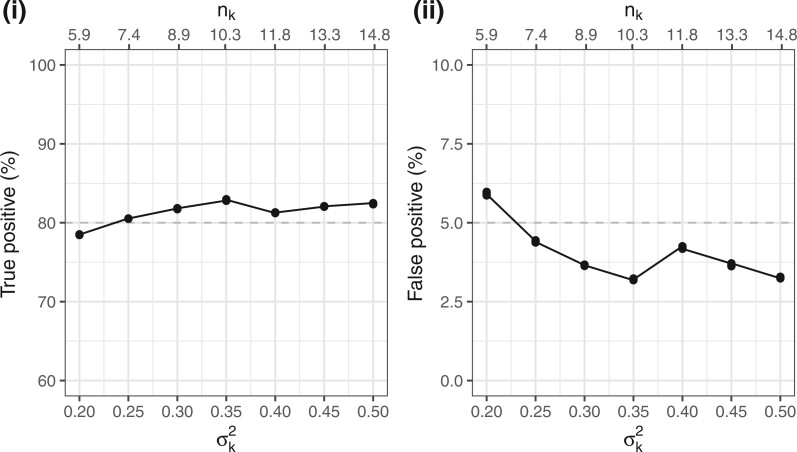
The true and false positive rates per subtrials $k = 1, \dots, 7$, summarized based on the 100 000 replicates of basket trials, wherein $X_{iEk}$ are generated from $N(-0.4, \sigma_k^2)$ and $N(0, \sigma_k^2)$ distributions for panels (i) and (ii), respectively. The dashed lines indicate the prespecified level of $\zeta = 0.80$ or $1-\eta = 0.05$.

Focusing on Scenarios 4–6 for the true positive and false positive rates at the subtrial levels, the proportions are not exactly 80$\%$ or 5$\%$ because of the simulation randomness. Additional simulations have been carried out for homoscedastic scenarios by varying the value of $\sigma_k^2$. Figure 3 shows (i) the subtrialwise sample sizes, $n_k$, determined based on our sample size formula in (2.11), and correspondingly, (ii) the subtrialwise true positive and false positive rates based on the simulated 100 000 replicates of basket trials. Each set of the additional simulations yield seven points (for $K=7$ subtrials), which congregate at the levels around $\zeta = 0.80$ or $1-\eta = 0.05$. In summary, the proposed methodology can lead to the control of error rates.

We have also performed a sensitivity analysis to understand the effect of misspecified values of $w_{qk}$. Table S2 of the Supplementary material available at *Biostatistics* online reveals that the proposed methodology is reasonably robust to the misspecification of $w_{qk}$. Nonetheless, care is needed when the value of $w_{qk}$ in the analysis deviates too far from that used in the design. When $w_{qk}$ is set to a larger value in the analysis than in the design (i.e., less borrowing is implemented than planned), a smaller percentage of trials conclude with a decisive decision. Whereas, a smaller value of $w_{qk}$ would yield a more informative posterior distribution, but this sometimes produces ambiguous conclusion of effectiveness or futility.

## 5. Discussion

The importance of choosing an appropriate sample size can never be overemphasized (Senn, 2007). Whilst basket trials have major infrastructural and logistical advantages, sophisticated statistical models are needed for the sample size planning to preserve the added efficiency. The most widely used approach to date is based on a Bayesian stand-alone analysis model, which does not support information sharing across subtrials with commensurate treatment effects. Consequently, the majority of basket trials recruit a higher sample size than required. This not only causes a waste of resources but could sometimes be unethical for exposing more patients than is necessary to a treatment that is yet to be fully approved (Altman, 1980). To realize the promise of basket trials, this article establishes a closed-form solution to the simultaneous determination of subtrial sample sizes. The simulation study shows that the proposed methodology allows for a smaller trial sample size wherever $0\leq w_{qk}<1$, without undermining the chance of detecting if there exists a clinically relevant difference between the experimental treatment and the control.

For deriving our sample size formulae, we adopted the Bayesian decision-making scheme elaborated by Whitehead *and others* (2008). Specifically, it involves two probability thresholds $\eta$ and $\zeta$ for reaching a decisive statement on the treatment’s effectiveness or futility. In our numerical illustration, we set $\eta = 0.95$ and $\zeta = 0.80$ because these probability quantities yield $n_k^0$, obtained based on the approach of no borrowing, comparable to the frequentist solution of sample sizes with $\alpha = 0.05$ and $\beta = 0.20$. Other choices may certainly be feasible: there is no conventional level to set these probability thresholds. In practice, these quantities might be difficult to justify: fixing $\eta$ to, for example, 0.95 might mean a considerable increase in sample size compared to 0.90. Since the sample sizes also depend on other parameters, we recommend the user generates plots for their cases following the pattern of our Figure S2 of the Supplementary material available at *Biostatistics* online.

Two sets of key parameters to implement the proposed methodology are the variances $\sigma_k^2$ and the levels of pairwise discounting $w_{qk}$. Similar to the widely used frequentist formulae (Chow *and others*, 2007), an increase in $\sigma_k^2$ would mean that larger sample sizes are needed to maintain the same level of precision in data. We have restricted our focus on known variances throughout since this is common in conducting clinical trials for most circumstances. Appropriate values for $\sigma_k^2$ to compute the subtrial sample sizes can be informed by pilot data or information from relevant investigations. If retaining $\sigma_k^2$ as unknown parameters, priors about their magnitude would be required. Subtrial sample sizes would then be sought by controlling the average property of posterior interval probabilities of $\theta_k$ with respect to 0 and $\delta$, since these nuisance parameters need to be integrated out from the posterior. Zheng *and others* (2022) derived sample size formulae with unknown $\sigma_k^2$ for a relevant context. Although different decision criteria were considered, one could follow their methodology to obtain the marginal posterior distributions, for which external information on $\sigma_k^2$ may further be incorporated. For wider applications, we have extended the proposed methodology for basket trials using a binary (in both the randomized controlled and single-arm settings) or time-to-event outcome; see Sections C–E of the Supplementary material available at *Biostatistics* online for the corresponding sample size formulae. In the meanwhile, we note there are limitations; for example, the censoring assumptions for time-to-event data are greatly simplified. We hope this work will stimulate further research within this Bayesian decision framework.

In the present work, data are supposed to be analyzed after the completion of all subtrials. However, in practice, certain subtrials may take much longer to complete recruitment due to low prevalence. One could (i) adopt a “first (subtrials) complete, first analyzed” principle, or (ii) alter the constraint for simultaneously solving $n_1, \dots, n_K$, for example, by making them proportional to the prevalence of respective target subpopulations, whilst maintaining an overall statistical power or decision accuracy. For strategy (i), more borrowing would be possible from subtrials that complete faster to those that complete slower. With strategy (ii), all subtrials may finish about the same time to yield a joint data analysis. We should note that it is not obvious if either strategy leads to a substantial increase in the total sample size.

When borrowing of information is permitted, a reduced sample size can be expected by setting $w_{qk} < 1$. The smaller the values of $w_{qk}$, the more borrowing is possible. The present methodology requires that these values be specified to reflect the pairwise (in)commensurability of subtrial data. This is especially feasible when a pilot study has been conducted. More details about the practical implementation, particularly the specification of parameters, are available in Section F of the Supplementary material available at *Biostatistics* online. Throughout, we have elaborated the methodology concerning a prespecified magnitude relating to the effect size, $\delta$, to find subtrial sample sizes. Extending the calculation to consider subtrial-specific effective sizes, say, $\delta_k$, is straightforward. A smaller value of $\delta_k$ would indicate that a larger $n_k$ is needed, if all other parameters are held fixed. For practical implementation, the user may substitute the corresponding argument (currently as a single value) by a vector in the openly available software.

Our sensitivity analysis in Section G of the Supplementary material available at *Biostatistics* online suggests the proposed methodology is reasonably robust against misspecification of $w_{qk}$. Nevertheless, when the values deviate too far from the truth, the resulting sample sizes would not reflect what is needed to achieve the trial’s objectives. One avenue for future research would therefore be developing methodology for sample size reassessment in basket trials. Practitioners may start with rather conservative choices of $w_{qk}$ assuming limited borrowing and re-estimate $w_{qk}$ using accumulating data from the ongoing trial at interims. This, however, bears the risk of inflated error rates, since the observed early-stage data are used to reassess the appropriate levels for borrowing. As rightly noted by the Associate Editor, one could incorporate the uncertainty in $w_{qk}$ for such updates within a Bayesian framework. Our subsequent work will investigate sample size reassessment in basket trials whilst avoiding the inflation of error rates. The proposed methodology may also be extended to enable mid-course adaptations. For instance, the basket trial will begin with a few subsets of interest and then restrict enrollment to the ones wherein patients benefit satisfactorily from the treatment based on an interim analysis. Boundaries for the early stopping of certain subsets must be carefully defined to protect the overall error rates. With a reduction in the number of subsets, the synthesis weights $p_{qk}$ should be updated to satisfy the constraint of $\sum_q p_{qk} = 1$ for the late stage(s).

## Supplementary Material

kxac033_Supplementary_Data

## Software

All statistical computing and analyses were performed using the software environment R version 4.0.3. Programming code for implementing the sample size formulae and reproducing the numerical results is available at https://github.com/haiyanzheng/BasketTrialsSSD.
