# Supplementary material for: Bayesian sample size determination in basket trials borrowing information between subsets
Source: Biostatistics. 2022 Aug 22;24(4):1000–16. doi: 10.1093/biostatistics/kxac033 (PMC11616727; doi:10.1093/biostatistics/kxac033)
Supplement: kxac033_Supplementary_Data [file kxac033_supplementary_data.pdf]

Supplementary Materials for:
Bayesian sample size determination in basket trials borrowing information between subsets

by Haiyan Zheng, Michael Grayling, Pavel Mozgunov, Thomas Jaki, James Wason

A. IMPACT OF SEVERAL KEY PARAMETERS ON THE SAMPLE SIZES

In this section, we illustrate how sample sizes change along with certain key parameters, such as the variances and w_{qk}, for a special case, where the basket trial has two subgroups only (i.e., $K = 2$). For illustration, we have set $s_{0k}^2 = 100$, $a_1 = b_1 = 2$, $a_2 = 54, b_2 = 3$ and $c_0 = 0.05$. Assuming $\sigma_1^2 = 0.587^2$, $\sigma_2^2 = 0.345^2$ (values were extracted from the SUMMIT trial which appears in Section 3.2 of the main paper) and $\delta = 0.4$, sample sizes are sought for the inferences about θ_k with $\eta = 0.95$ and $\zeta = 0.80$. As expected, the variance σ_k^2 is a key factor for the determination of the corresponding n_k. More precisely, sample size of subtrial 1 is generally greater than that of subtrial 2 when holding other parameters fixed.

Figure S1 confirms that the sample size of subtrial 1 (subtrial 2) additionally relies on w_{21} (w_{12}) which controls the degree of borrowing from subtrial 2 (subtrial 1): the n_ks in the same row within the subtrial 1 plot and those in the same column within the subtrial 2 plot remain equal by nearest integer for most cases. The only exceptions are the row with $w_{21} = 0$ in the subtrial 1 plot and the column with $w_{12} = 0$ in the subtrial 2 plot, where such variation is often trivial. We note this is caused by the use of Newton's method for solving the nonlinear equations. Figure S1 also indicates that an increase or decrease in w_{qk} does not lead to linear change on the sample size of subtrial k, but with [0, 0.3] being a more sensitive range than (0.3, 1]. Substantial saving in the total sample size would be achieved if setting w_{21} and w_{12} to small values, for example, both as 0. Focusing on the third plane, the number at the bottom left goes larger when moving towards the top right.

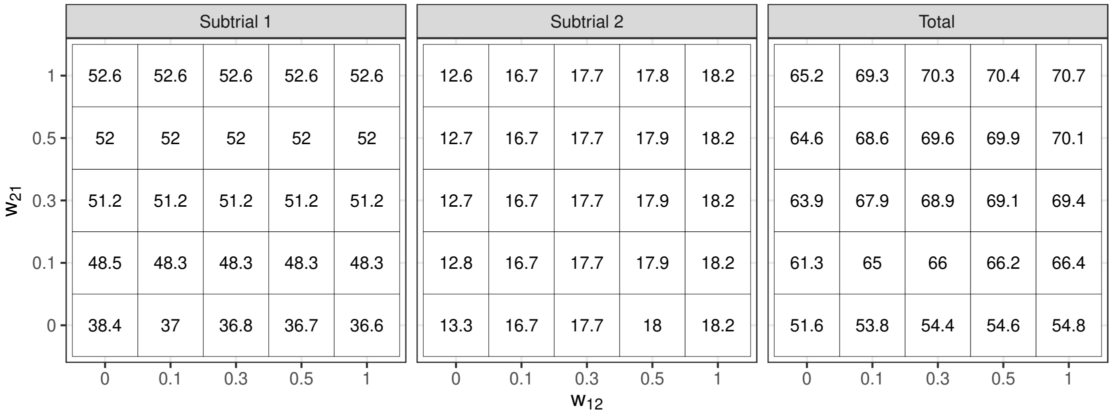

Figure S1: Bayesian sample sizes of the respective subtrials and entire basket trial with $K = 2$, given various levels of incommensurability.

Furthermore, n_1 and n_2 computed here based on the proposed sample size formula are bounded

by 52.6 (resulting from $w_{21} = 1$) and 18.2 (resulting from $w_{12} = 1$), respectively. These are close to $n_1^0 = 53.2$ and $n_2^0 = 18.4$, as obtained from the approach of no borrowing.

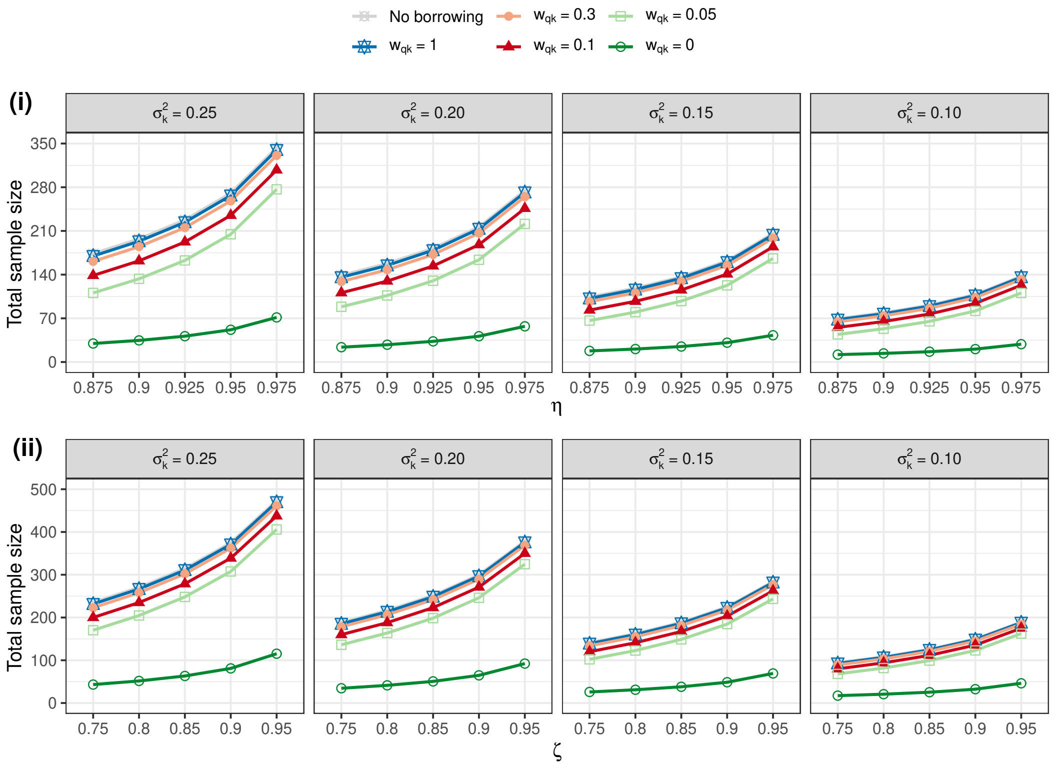

Figure S2: Total sample size targeting different levels of η and ζ. In panel (i), ζ is fixed at 0.80; while in panel (ii), η is fixed at 0.95.

We now explore the behaviour of our Bayesian sample size formulae (when setting $0 \leq w_{qk} \leq 1$) in comparison to that of the approach of no borrowing. Focusing on homoskedastic cases (i.e., σ_k^2 remain identical across subtrials), Figure S2 shows that the trial sample size increases as the probability thresholds, η or ζ, increase. This is unsurprising, since a larger probability threshold would mean a more informative posterior distribution for θ_k to reach a decisive conclusion.

We also observe that sample size reduction is possible by setting a small value for w_{qk}. More specifically, $\sum n_k$ drops quickly when reducing w_{qk} from 0.1 to 0 in both panels (i) and (ii). Looking across the plots of the same panel, the larger (smaller) the variances, the greater (less) the sample size would be required.

We then visualise the required sample sizes for different standardised effect sizes, δ/σ_k^2, in Figure S3. We set $\sigma_k^2 = 0.25$ throughout this illustration and $\delta = 0.60, 0.55, 0.50, 0.45, 0.40, 0.35, 0.30$. Unsurprisingly, larger sample sizes are required to detect a smaller standardised effective size. Figure S3 also suggest that a value close to 0 for w_{qk} results in substantial saving of sample size.

B. SIMULATION SCENARIOS AND ADDITIONAL RESULTS

Table S1 lists the six scenarios that have been used in our simulation study of the main paper. Additional simulation results based on a sensitivity analysis will also be presented in this section.

Additional simulations have been performed to illustrate the proposed methodology controls

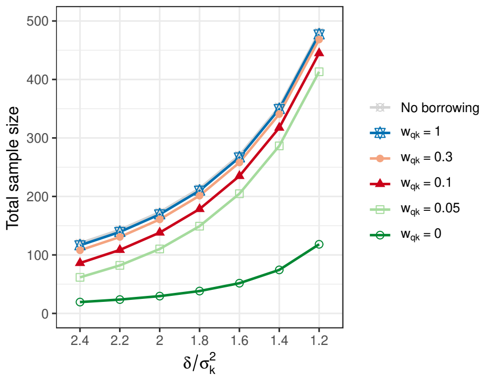

Figure S3: Total sample size, as a function of the standardised effect size, δ/σ_k^2, by the proposed methodology and the approach of no borrowing, respectively, varying $w_{qk} = 0, 0.05, 0.1, 0.3, 1$.

Table S1: Simulation scenarios depicted as outcome distributions for the experimental treatment, $N(\mu_{Ek}, \sigma_k^2)$, $k = 1, \ldots, 7$. The corresponding outcome distribution for the control is $N(0, \sigma_k^2)$.

		Subtrial						
		$k=1$	$k=2$	$k=3$	$k=4$	$k=5$	$k=6$	$k=7$
Scenario 1	μ_{Ek}	-0.489	0.226	-0.181	0.293	0.329	-0.275	-0.136
	σ_k^2	0.345	0.119	0.144	0.120	0.118	0.154	0.154
Scenario 2	μ_{Ek}	-0.289	-0.226	-0.281	-0.293	-0.329	-0.275	-0.236
	σ_k^2	0.345	0.119	0.144	0.120	0.118	0.154	0.154
Scenario 3	μ_{Ek}	-0.289	-0.226	-0.281	-0.293	-0.329	-0.275	-0.236
	σ_k^2	0.300	0.300	0.300	0.300	0.300	0.300	0.300
Scenario 4	μ_{Ek}	-0.400	-0.400	-0.400	-0.400	-0.400	-0.400	-0.400
	σ_k^2	0.300	0.300	0.300	0.300	0.300	0.300	0.300
Scenario 5	μ_{Ek}	-0.289	0	-0.181	0	0	-0.275	0
	σ_k^2	0.345	0.119	0.144	0.120	0.118	0.154	0.154
Scenario 6	μ_{Ek}	0	0	0	0	0	0	0
	σ_k^2	0.300	0.300	0.300	0.300	0.300	0.300	0.300

the error rates at desired levels for each subtrial. Figure S4 visualises the accuracy of decision under two new mixed null scenarios, i.e., Scenario A with $\theta_1 = \theta_3 = \theta_6 = -0.4$ and Scenario B with $\theta_1 = \theta_3 = \theta_6 = -0.5$ along with $\theta_2 = \theta_4 = \theta_5 = \theta_7 = 0$ in both. The variances are set the same as those displayed in Scenario 5 above. As we can read from the figure, assuming the desired effect size $\delta = -0.4$, the percentage of simulations incorrectly declaring E as efficacious for subtrials with $\theta_k = 0$ is maintained below 5% using the proposed methodology.

C. EXTENDED APPLICATION TO USING A BINARY OUTCOME

When a binary outcome is used in randomised basket trials, the primary interest is to compare the response rates on the respective treatments, denoted by ρ_{Ek} and ρ_{Ck}, in each subtrial $k = 1, \ldots, K$. In this section, we adapt the proposed methodology to enable borrowing of information in terms of log-odds ratios, which are approximately normally distributed [1].

Let n_k be the subtrial sample size, for $k = 1, \ldots, K$. The log-odds ratio based on the individual

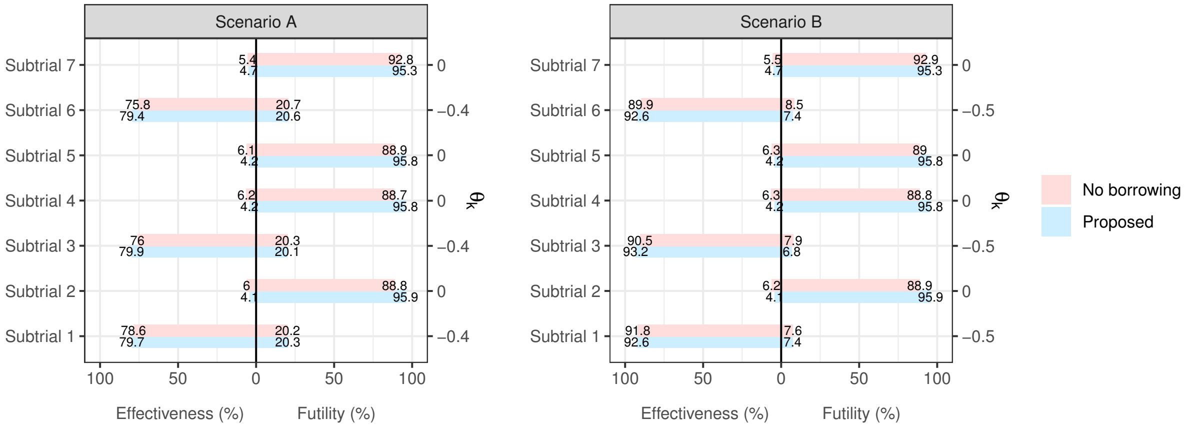

Figure S4: Percentage of (sub)trials that conclude E is efficacious (the left half of each plot) or not better than C by $\delta = -0.4$ under two new scenarios.

subtrial data is

$$\log(\hat{OR}_k) \dot{\sim} N\left(\log\left(\frac{\rho_{Ek}(1-\rho_{Ck})}{(1-\rho_{Ek})\rho_{Ck}}\right), \frac{1}{n_k}\left(\frac{1}{\rho_{Ek}} + \frac{1}{1-\rho_{Ek}} + \frac{1}{\rho_{Ck}} + \frac{1}{1-\rho_{Ck}}\right)\right).$$

We further let $\theta_k = \log[(\rho_{Ek}(1-\rho_{Ck}))/((1-\rho_{Ek})\rho_{Ck})]$. Following the methodology proposed in the main paper, we represent the complementary subtrial data in commensurate priors and place a two-component Gamma mixture prior on each commensurate parameter. This gives

$$\theta_k \mid \boldsymbol{x}_k, \boldsymbol{x}_{(-k)} \dot{\sim} N\left(d_{\theta_k}, \left(\frac{1}{\sum_q p_{qk}^2 \xi_{qk}^2} + n_k\left(\frac{1}{\rho_{Ek}(1-\rho_{Ek})} + \frac{1}{\rho_{Ck}(1-\rho_{Ck})}\right)^{-1}\right)^{-1}\right), \tag{S1}$$

with

$$\xi_{qk}^2 = \left(\frac{1}{s_{0q}^2} + n_q\left(\frac{1}{\rho_{Eq}(1-\rho_{Eq})} + \frac{1}{\rho_{Cq}(1-\rho_{Cq})}\right)^{-1}\right)^{-1} + \frac{w_{qk}b_1}{a_1 - 1} + \frac{(1-w_{qk})b_2}{a_2 - 1}.$$

Applying the same decision criterion with a clinically meaningful effect size, denoted by δ, the subtrial sample sizes can be found so that the K nonlinear equations hold simultaneously:

$$n_k \geq \left[\left(\frac{z_\eta + z_\zeta}{\delta}\right)^2 - \frac{1}{\sum_q p_{qk}^2 \xi_{qk}^2}\right]\left(\frac{1}{\rho_{Ek}(1-\rho_{Ek})} + \frac{1}{\rho_{Ck}(1-\rho_{Ck})}\right), \qquad \forall k = 1, \ldots, K. \tag{S2}$$

D. EXTENDED APPLICATION TO USING A TIME-TO-EVENT OUTCOME

We now consider extending the proposed sample size formulae to design randomised basket trials with a time-to-event outcome. For simplicity, we follow George and Desu [2] to assume that the event time, denoted by T_{ijk}, has an exponential distribution:

$$T_{ijk} \sim \text{Exp}(\pi_{jk}),\ i = 1, \ldots, n_k;\ j = E, C;\ k = 1, \ldots, K,$$

where the rate parameter $\pi_{jk} > 0$. Denote the average event time on treatment group j by $\bar{T}_{jk}$ and the number of events by D_{jk} in subtrial $k = 1, \ldots, K$. By the central limit theorem, we know

$$\bar{T}_{jk} \dot{\sim} N\left(\frac{1}{\pi_{jk}}, \frac{1}{D_{jk}\pi_{jk}}\right).$$

Using the delta method, we obtain

$$\log(\bar{T}_{jk}) \dot{\sim} N\left(-\log(\pi_{jk}), \frac{1}{D_{jk}}\right),$$

and further that

$$\log\left(\frac{\bar{T}_{Ek}}{\bar{T}_{Ck}}\right) \dot{\sim} N\left(\log(\frac{\pi_{Ck}}{\pi_{Ek}}), \frac{1}{D_{Ek}} + \frac{1}{D_{Ck}}\right).$$

Likewise, we let $\theta_k = \log(\frac{\pi_{Ck}}{\pi_{Ek}})$ and follow the proposed Bayesian methodology to enable borrowing of information between subtrials. This gives

$$\theta_k \mid \boldsymbol{x}_k, \boldsymbol{x}_{(-k)} \dot{\sim} N\left(d_{\theta_k}, \left(\frac{1}{\sum_q p_{qk}^2 \xi_{qk}^2} + \left(\frac{1}{D_{Ek}} + \frac{1}{D_{Ck}}\right)^{-1}\right)^{-1}\right), \tag{S3}$$

with

$$d_{\theta_k} = \frac{\frac{1}{D_{Ek}} + \frac{1}{D_{Ck}}}{\sum_q p_{qk}^2 \xi_{qk}^2 + \frac{1}{D_{Ek}} + \frac{1}{D_{Ck}}} \cdot \sum_q p_{qk}\lambda_{qk} + \frac{\sum_q p_{qk}^2 \xi_{qk}^2}{\sum_q p_{qk}^2 \xi_{qk}^2 + \frac{1}{D_{Ek}} + \frac{1}{D_{Ck}}} \cdot \log\left(\frac{\bar{t}_{Ek}}{\bar{t}_{Ck}}\right)$$

and

$$\xi_{qk}^2 = \left(\frac{1}{s_{0q}^2} + \left(\frac{1}{D_{Eq}} + \frac{1}{D_{Cq}}\right)^{-1}\right)^{-1} + \frac{w_{qk}b_1}{a_1 - 1} + \frac{(1 - w_{qk})b_2}{a_2 - 1}.$$

Applying the same decision criterion as the main paper, the subtrial sample sizes can be found according to

$$\frac{D_{Ek}D_{Ck}}{D_{Ek} + D_{Ck}} \geq \left(\frac{z_\eta + z_\zeta}{\delta}\right)^2 - \frac{1}{\sum_q p_{qk}^2 \xi_{qk}^2}.$$

Equivalently,

$$D_k \geq \frac{1}{R_k(1 - R_k)}\left[\left(\frac{z_\eta + z_\zeta}{\delta}\right)^2 - \frac{1}{\sum_q p_{qk}^2 \xi_{qk}^2}\right], \tag{S4}$$

where $D_k = D_{Ek} + D_{Ck}$ denotes the total number of events required per subtrial k, and R_k is the randomisation ratio to the experimental treatment E.

E. EXTENDED APPLICATION TO SINGLE-ARM SETTINGS WITH A BINARY OUTCOME

As one reviewer noted, basket trials are frequently conducted within a phase II oncology setting. Suppose that patients with a common feature (e.g., genomic aberration or clinical symptom) are enrolled to a basket trial with K subsets; each subset represents a subtype of the disease. Within each trial subset (i.e., 'subtrial' for short) $k = 1, \ldots, K$, patients are treated by a new treatment (labelled E) and observed to be either a 'responder' or 'non-responder' according to predefined criteria relating to the tumour shrinkage. Let Y_k denote the number of responders and n_k the number of patients per subtrial k. Thus, $\mathbb{E}(Y) = n_k\rho_k$ and $\text{Var}(Y) = n_k\rho_k(1 - \rho_k)$, where ρ_k is the

subtrial-specific response rate. By the delta method, the estimator of log-odds has an asymptotic normal distribution of

$$\log\left(\frac{\hat{\rho}_k}{1-\hat{\rho}_k}\right) \dot{\sim} N\left(\log\left(\frac{\rho_k}{1-\rho_k}\right), \frac{1}{n_k\rho_k(1-\rho_k)}\right)$$

Letting $\theta_k = \log(\rho_k/(1-\rho_k))$, we follow the proposed methodology to specify commensurate priors for θ_k, based on the complementary subtrial data, and place a two-component Gamma mixture prior on each commensurate parameter. This then leads to

$$\theta_k \mid \boldsymbol{x}_k, \boldsymbol{x}_{(-k)} \dot{\sim} N\left(d_{\theta_k}, \left(\frac{1}{\sum_q p_{qk}^2 \xi_{qk}^2} + n_k\rho_k(1-\rho_k)\right)^{-1}\right), \tag{S5}$$

with

$$\xi_{qk}^2 = \left(\frac{1}{s_{0q}^2} + n_q\rho_q(1-\rho_q)\right)^{-1} + \frac{w_{qk}b_1}{a_1-1} + \frac{(1-w_{qk})b_2}{a_2-1}.$$

Applying the same decision criterion, the subtrial sample sizes can be found to satisfy the K non-linear equations simultaneously:

$$n_k \geq \frac{1}{\rho_k(1-\rho_k)}\left[\left(\frac{z_\eta + z_\zeta}{\delta}\right)^2 - \frac{1}{\sum_q p_{qk}^2 \xi_{qk}^2}\right]. \tag{S6}$$

F. PRACTICAL IMPLEMENTATION OF THE PROPOSED SAMPLE SIZE FORMULAE

Figure S5 guides through the specification of parameters (specific to the design and data type) to implement the proposed Bayesian sample size formulae for basket trials, wherein borrowing of information is enabled wih $w_{qk} < 1$.

As noted, setting $\eta = 0.95$ and $\zeta = 0.80$ creates a resemblance to the frequentist formulation of the problem in situations of no borrowing (with all $w_{qk} = 1$). The user may modify these probability thresholds for their own case. Raising the level of η or ζ would mean an increase in the sample size. We recommend the user to visualise such changes following the pattern of Figure S2. This would lead to effective communication with the stakeholder for finding sample sizes that are both affordable and sensible for estimating the effect size. In the randomised, two-arm settings, we have considered equal randomisation throughout by setting $R_1 = \cdots = R_K = 0.5$. The user may change the allocation ratio for their context.

Having chosen a design and an outcome to evaluate the clinical benefit, the user should seek information regarding (i) plausible response rate (ρ_k or ρ_{jk} for $j = E, C$, if using a binary outcome) or (ii) variability of responses (σ_k^2, if using a continuous outcome), both specific to subtrial $k = 1, \ldots, K$, along with a clinically meaningful effect size (denoted by δ', δ^*, δ or $\delta^\dagger$ in Figure S5). This would be substantially easier if pilot data or historical information is available.

Specification of w_{qk} to correctly reflect the pairwise incommensurability might be challenging in practice. This should best be informed by pilot data or existing information from relevant investigation. Following the derivation of the sample size formulae in Section 2 of the main paper, values for w_{qk} can be chosen independently of the variances, σ_k^2, $k = 1, \ldots, K$. For continuous data, however, values of σ_k^2 and w_{qk} can both be based on existing information from, e.g., a pilot study or preceding trial. We have exemplified this in Section 3.2 of the main paper, where the Hellinger distance is used to measure the discrepancy between the outcome distributions. The latter can

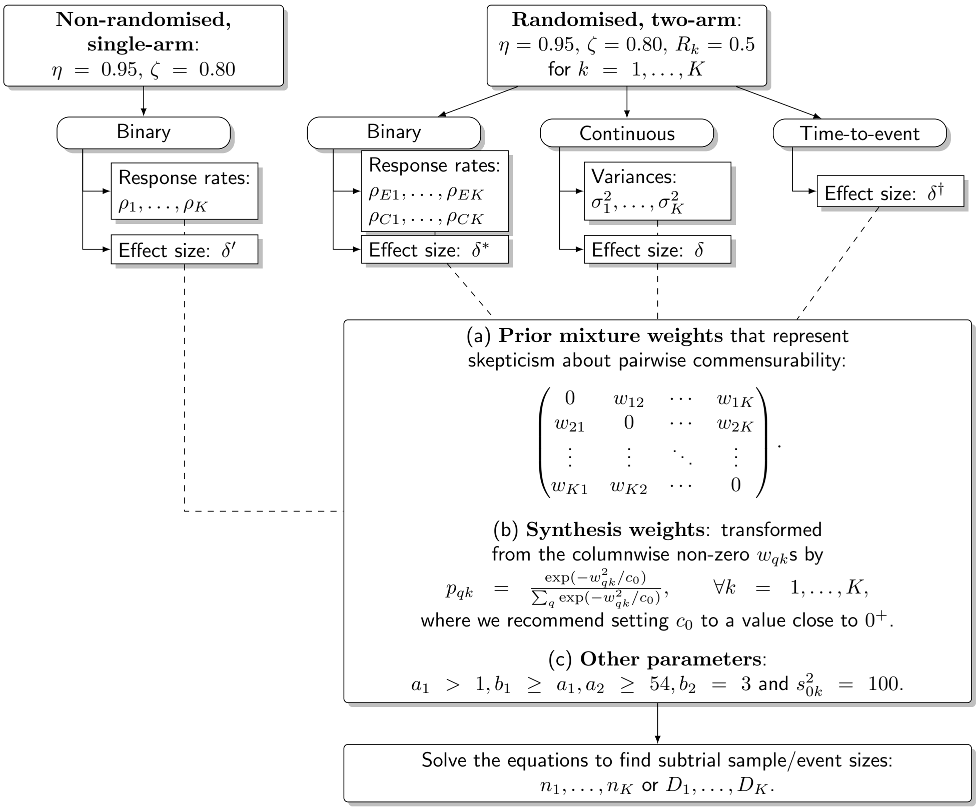

Figure S5: A roadmap for parameter specification in the proposed sample size formulae to design basket trials that may adopt a (i) non-randomised, single-arm, or (ii) randomised, two-arm setting in the respective subsets. Specific to the data type, the effect size (denoted by δ', δ^*, δ or $\delta^\dagger$) is on a scale of log-odds, log-odds ratio, mean difference or log-hazard ratio, respectively.

be assumed with available data from a preceding trial (i.e., the SUMMIT trial in Section 3.2). For basket trials using a binary or time-to-event outcome, one may likewise compute the Hellinger distance between asymptotic normal distributions of the subtrial-specific log-odds, log-odds ratio or log-hazard ratio.

In these circumstances, the assumed values for response rates or variances affect the levels of w_{qk}, and the impact on the corresponding subtrial sample sizes may thus be of interest. We refer the reader to Table 1 of Section 4, which expands the illustration of sample size determination with various scenarios. Specifically, the variances in subsets 2 – 7 are increased from Scenario 1 (used in Section 3.2) to Scenario 3, which gives a different set of values for w_{qk}; see Figure 2 of the main paper for the visualisation. The resulting sample size is then an outcome of the increased variances and declined levels of pairwise incommensurability (i.e., increased amount of borrowing): the sample sizes $n_2, \ldots, n_7$ based on the proposed approach increase from Scenario 1 to Scenario 3, whereas the magnitude of such increase is not as large as that of $n_2^0, \ldots, n_7^0$ based on the approach of no borrowing; please see Table 1 of the main paper for this.

For the hyperparameters, the only constraint is $a_1, a_2 > 1$. As shown with illustrative examples in the main paper, we recommend setting these hyperparameters to give a small prior mean (e.g.,

$a_1/b_1 \leq 1$) by the first component and a large prior mean (e.g., $a_2/b_2 \geq 10$) by the second.

G. CONSEQUENCE OF PARAMETER MISSPECIFICATION FOR w_{qk}

Following the specification of w_{qk} outlined in Section 3.2 of the main paper, we compute the Hellinger distance between any pair of $N(\mu_{Ek}, \sigma_k^2)$. Thus, we obtain

$$\begin{pmatrix} w_{11} & w_{12} & \cdots & w_{17} \\ w_{21} & w_{22} & \cdots & w_{27} \\ \vdots & \vdots & \cdots & \vdots \\ w_{71} & w_{72} & \cdots & w_{77} \end{pmatrix} = \begin{pmatrix} 0 & 0.539 & 0.300 & 0.571 & 0.591 & 0.246 & 0.312 \\ 0.539 & 0 & 0.384 & 0.068 & 0.105 & 0.457 & 0.342 \\ 0.300 & 0.384 & 0 & 0.439 & 0.470 & 0.087 & 0.044 \\ 0.571 & 0.068 & 0.439 & 0 & 0.037 & 0.508 & 0.397 \\ 0.591 & 0.105 & 0.470 & 0.037 & 0 & 0.537 & 0.429 \\ 0.246 & 0.457 & 0.087 & 0.508 & 0.537 & 0 & 0.125 \\ 0.312 & 0.342 & 0.044 & 0.397 & 0.429 & 0.125 & 0 \end{pmatrix},$$

for Scenario 1 and all $w_{qk} = 0$ for Scenario 6. When the same matrix of w_{qk} is used to analyse the trial, the operating characteristics of the proposed design are as visualised in plots (a) and (f) of Figure 3 of the main paper, respectively. For our interest in the impact of misspecification of w_{qk}, we assume that three 7×7 matrix, wherein $w_{qk} = 0.1, 0.3, 0.5$ (for $q \neq k$) along with $w_{kk} = 0$, $k = 1, \ldots, 7$, would be applied in the Bayesian analysis. In the following sensitivity analysis, we simulate 100,000 replicates of the basket trial with the same parameter configuration as that of the main simulation study.

Table S2: Percentage of (sub)trials that conclude effectiveness or futility of E in situations where the values of w_{qk} are correctly or incorrectly specified.

			Subtrial						
			$k=1$	$k=2$	$k=3$	$k=4$	$k=5$	$k=6$	$k=7$
Scenario 1	True w_{qk}	Effectiveness (%)	91.5	0.1	30.2	0	0	51.6	21.1
		Futility (%)	8.5	99.9	69.8	100	100	48.4	78.9
	$w_{qk} = 0.1$	Effectiveness (%)	88.8	0.2	25.6	0	0	46.1	17.5
		Futility (%)	14.0	99.9	76.4	100	100	57.8	84.3
	$w_{qk} = 0.3$	Effectiveness (%)	90.5	0.2	27.7	0	0	49.0	19.1
		Futility (%)	10.0	99.8	70.9	99.9	100	50.6	79.9
	$w_{qk} = 0.5$	Effectiveness (%)	90.8	0.2	28.2	0	0	49.7	19.5
		Futility (%)	9.3	99.8	69.5	99.9	100	49.1	78.9
Scenario 6	True w_{qk}	Effectiveness (%)	3.7	3.7	3.7	3.7	3.7	3.6	3.6
		Futility (%)	96.3	96.3	96.3	96.3	96.3	96.4	96.4
	$w_{qk} = 0.1$	Effectiveness (%)	2.6	2.6	2.7	2.7	2.7	2.5	2.7
		Futility (%)	73.8	74.2	73.8	74.1	73.8	73.8	73.8
	$w_{qk} = 0.3$	Effectiveness (%)	4.2	4.1	4.3	4.2	4.2	4.1	4.3
		Futility (%)	64.8	65.1	64.9	65.1	64.8	64.7	64.8
	$w_{qk} = 0.5$	Effectiveness (%)	4.7	4.7	4.8	4.8	4.7	4.7	4.8
		Futility (%)	62.6	62.9	62.7	62.9	62.7	62.5	62.6

Table S2 shows that when true values of w_{qk} are used in the analysis, all of the simulated trials will be assigned a decisive subtrialwise conclusion (100% = Effectiveness% + Futility%). When w_{qk} is specified as a greater value in the analysis than that used in the design (meaning that the amount of borrowing is attenuated), a smaller proportion of trials will reach a decisive decision

making. This is evident by the results for Scenario 6 in Table S2 at the rows of $w_{qk} = 0.1, 0.3, 0.5$ (all being > 0 which is the value of w_{qk} used in the design). The more the values deviate from the designated level, the fewer trials could be concluded correctly on the futility of treatment in Scenario 6. By contrast, when w_{qk} would be specified to a smaller value, some trials may produce an ambiguous decision on effectiveness or futility; see, for example, $k = 1$ in Scenario 1 with all $w_{qk} = 0.1$ which are consistently lower than the values used for the design. When the magnitude of deviation is small, the impact of misspecification is trivial: the percentages for $k = 7$ in Scenario 1 with $w_{qk} = 0.3$ are comparable to those with true w_{qk} used in the analysis.

Finally, we would reemphasise that the change of w_{qk} does not lead to a linear increase or decrease in the corresponding sample size. As Figure 1 in the main paper illustrates, the region bounded by 0 and 0.3 tends to be more sensitive to the other half, particularly from 0.5 to 1. Therefore, attention would be needed for the misspecification of w_{qk}, if the true values are {*either*} *within the sensitive region* {or} the deviated across the region (e.g., setting $w_{qk} = 0.55$ in the design yet $w_{qk} = 0.25$ in the analysis, or the other way around).

References

1. Agresti, A. (2003). *Categorical Data Analysis, Second Edition*. Wiley Series in Probability and Statistics, Hoboken. NJ: Wiley.

2. George, S. L. and Desu, M. M. (1974). Planning the size and duration of a clinical trial studying the time to some critical event. *Journal of Chronic Diseases* **27**(1), 15 – 24.
